# Investigation on the Overshoot of Transient Open-Circuit Voltage in Methylammonium Lead Iodide Perovskite Solar Cells

**DOI:** 10.3390/ma11122407

**Published:** 2018-11-29

**Authors:** Chunhai Li, Longfeng Lv, Liang Qin, Lijie Zhu, Feng Teng, Zhidong Lou, Zhenbo Deng, Yufeng Hu, Qiuhong Cui, Yanbing Hou

**Affiliations:** Key Laboratory of Luminescence and Optical Information, Ministry of Education, Beijing JiaoTong University, Beijing 100044, China; 12118408@bjtu.edu.cn (C.L.); 11118405@bjtu.edu.cn (L.L.); 14118430@bjtu.edu.cn (L.Q.); 12118410@bjtu.edu.cn (L.Z.); fteng@bjtu.edu.cn (F.T.); zhdlou@bjtu.edu.cn (Z.L.); zbdeng@bjtu.edu.cn (Z.D.); yfhu@bjtu.edu.cn (Y.H.); qiuhcui@bjtu.edu.cn (Q.C.)

**Keywords:** overshoot, transient photovoltage, perovskite solar cell, ionic migration

## Abstract

Although the performance of hybrid organic-inorganic perovskite solar cells (PSCs) is encouraging, the detailed working principles and mechanisms of PSCs remain to be further studied. In this work, an overshoot phenomenon of open-circuit voltage (*V_oc_*) was observed when the illumination light pulse was switched off. The evolution of the *V_oc_* overshoot was systematically investigated along with the intensity and the width of the light pulse, the background illumination, and pretreatment by different bias. Based on the experimental results, we could conclude that the *V_oc_* overshoot originated from carrier motion against carrier collection direction, which happened at the ionic-accumulation-induced band bending areas near the interfaces between the perovskite active layer and the two carrier transport layers. The investigation on the *V_oc_* overshoot can help us to better understand ionic migration, carrier accumulation, and recombination of PSCs under open-circuit conditions.

## 1. Introduction

Hybrid organic-inorganic perovskite solar cells (PSCs) have attracted a lot of attention due to their rapidly increased power conversion efficiency (PCE) from 3.8% [1] to 23.2% [2]. However, the detailed working principles and mechanisms of PSCs are still not clear. The possible reasons for this phenomenon include ionic migration [3,4,5,6], ferroelectricity [7,8], charge trapping/detrapping [9,10], and capacitive effects [11].

Among these mechanisms, ionic migration has been considered to be the main reason for the hysteresis behavior in PSCs [12]. Therefore, much attention has been focused on the role of ions/vacancies driven by the built-in, photogenerated, and external electric fields [5,6]. To minimize hysteresis, some strategies have been proposed, such as preparing the perovskite crystal with larger grain size [11], using mesoporous titanium dioxide (m-TiO_2_) layers as the electron transport layer (ETL) [13], and using fullerene as the passivation layer to modify TiO_2_ [14] or the perovskite layer [15]. However, the method to avoid hysteresis with fullerene derivative as passivation layer does not seem to work all the time. In Khadka et al.’s work, PSCs with fullerene derivatives still showed hysteresis more or less [16]. Meanwhile, even some hysteresis-free PSCs still showed hysteresis behavior at low temperature [17]. Calado et al. found that ionic migration also existed in PSCs with minimal hysteresis [18].

Transient photovoltage (TPV), proposed as a tool to study the recombination of charge carriers in semiconductor diodes [19,20,21], has been widely used to investigate charge generation and recombination processes in solar cells [22,23,24]. In recent years, several groups have studied the dynamic characteristics and hysteresis of PSCs by TPV measurements. For example, Baumann et al. found that the recombination dynamics in all time regimes were dependent on the starting illumination intensity [25]. O’Regan et al. proved that hysteresis was not caused by the change of recombination rate and charge separation efficiency [26].

In this report, we performed TPV measurement on methylammonium lead iodide (MAPbI_3_) PSCs with rutile titanium dioxide (R-TiO_2_) nanocrystals as the ETL and observed an unpredictable overshoot of *V_oc_*, instead of a descent as expected, when the illumination was switched off. This phenomenon was never observed in our previous work on organic solar cells (shown in Figure S1). O’Regan et al. had also observed a similar phenomenon, but they had attributed the overshoot to noise [26]. After investigating the evolution of the overshoot with various kinds of pulse light illumination, we suggest that the observed *V_oc_* overshoot resulted from ionic migration.

## 2. Experimental Section

### 2.1. Materials

Lead iodide (PbI_2_), *tert*-butylpyridine (*t*-BP), and lithium-bis(trifluoromethanesulfonyl)imide (Li-TFSI) was purchased from Sigma-Aldrich, (Shanghai, China). Methylammonium iodide (MAI) was purchased from Dyesol, (Queanbeyan, Australia). 2,2′,7,7′-tetrakis-(*N*,*N*-di-4-methoxyphenylamine)-9,9′-spirobifluorene (spiro-OMeTAD) was purchased from 1-Material, (Dorval, Canada). All the other chemicals were purchased from Alfa Aesar (Shanghai, China) and were used as received.

### 2.2. Preparation of R-TiO_2_ Nanocrystals

The ligand-free R-TiO_2_ nanocrystals were made using an earlier reported method [27]. First, 2 mL titanium trichloride (TiCl_3_, 15.0–20.0% basis in 30% HCl) and 1 mL stannic chloride (SnCl_4_) aqueous solution (0.5 M) were added into 60 mL ethanol. Then, the mixed solution was placed in a drying oven at 75 °C for 8 h to get R-TiO_2_ nanocrystals suspension. After being purified by ethanol and dispersed into water, a 12 mg/mL R-TiO_2_ nanocrystal aqueous solution was obtained.

### 2.3. MAPbI_3_ PSC Fabrication

The indium tin oxide (ITO)-coated glass substrates were cleaned by a neutral cleaning solution and then given an ultrasonic bath in deionized water, acetone, and isopropyl alcohol (IPA) for 20 min each. After 3 min ultraviolet–ozone treatment, TiO_2_ nanocrystal layer with the thickness of ~30 nm was spin-coated on the ITO-coated glass substrates, followed by drying on a hotplate at 100 °C in the air and at 120 °C in a nitrogen-filled glovebox for 15 min each. The device fabrication steps were all carried out in the glovebox. We used a previously reported two-step spin-coating procedure [28] to form a MAPbI_3_ layer. The PbI_2_ solution was prepared by dissolving 461 mg PbI_2_ in 1 mL *N*,*N*-dimethylformamide (DMF) under stirring at 70 °C overnight. The PbI_2_ solution was spin-coated on TiO_2_ film at 3000 rpm for 15 s followed by drying at 70 °C and cooling to room temperature. Next, 150 μL of MAI solution (12 mg in 1mL IPA) was loaded on the PbI_2_-coated substrate and kept still for 40 s, then spun at 9000 rpm for 3 s and annealed at 90 °C for 30 min. The spiro-OMeTAD solution was prepared by dissolving 68 mg spiro-OMeTAD in 950 μL chlorobenzene with 28 μL *t*-BP and 17.5 μL Li-TFSI solution (520 mg Li-TFSI in 1 mL acetonitrile) as additives. This solution was spin-coated on the perovskite film as hole transport layer (HTL) at 5000 rpm for 50 s. A ~10 nm molybdenum oxide (MoO_3_) layer and a ~200 nm Ag electrode were finally evaporated in a vacuum chamber with a pressure of 3 × 10^−5^ Pa, successively. The effective electrode area was 4.5 mm^2^, controlled by a mask.

## 3. Results and Discussion

**Figure 1:**
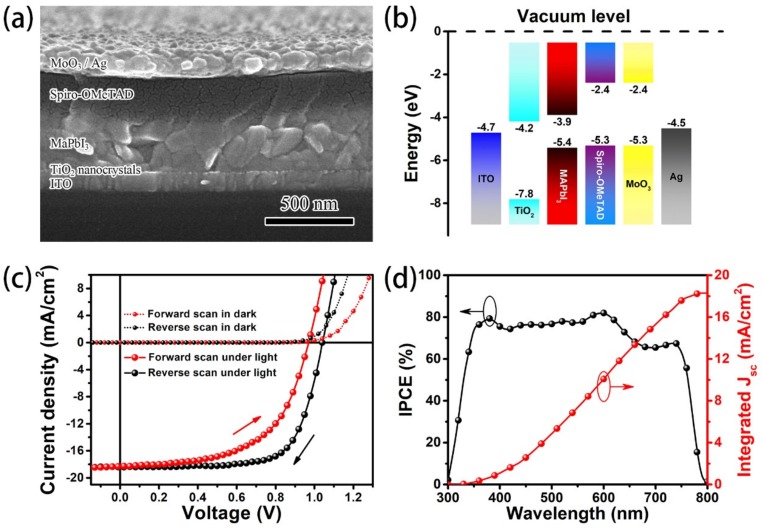
(**a**) Cross-sectional SEM image, (**b**) energy level diagram, (**c**) current density-voltage *(J-V*) characteristics, and (**d**) dependence of photon-to-current conversion efficiency (IPCE) on wavelength and the integrated short-circuit current density (*J_sc_*) of the perovskite solar cells (PSC) device.

The PSC device structure was ITO/TiO_2_/MAPbI_3_/spiro-OMeTAD/MoO_3_/Ag. A cross-sectional image of the PSC taken by Hitachi (S-4800, Tokyo, Japan) field emission scanning electron microscope (SEM) is shown in Figure 1a. The surface SEM image of the perovskite layer is shown in Figure S2. The energy level diagram is shown in Figure 1b. The current density–voltage (*J-V*) characteristics of PSCs were measured in the nitrogen atmosphere with a computer-controlled Keithley 6430 source measure unit under 100 mW/cm^2^ illumination generated by the AM 1.5 G solar simulator (XEC-301S, SAN-EI Electric, Osaka, Japan). The light intensity was calibrated by a silicon detector. We scanned the sample from 2 to −0.5 V (reverse scan) and then scanned it in the opposite direction (forward scan) continuously. The *J-V* characteristics of the device are shown in Figure 1c and Table S1. The incident photon-to-current conversion efficiency (IPCE) was measured by Zolix Solar Cell Scan 100, Beijing, China. The IPCE curve and the integrated short-circuit current density (*J_sc_*) of the PSC device are shown in Figure 1d.

**Figure 2:**
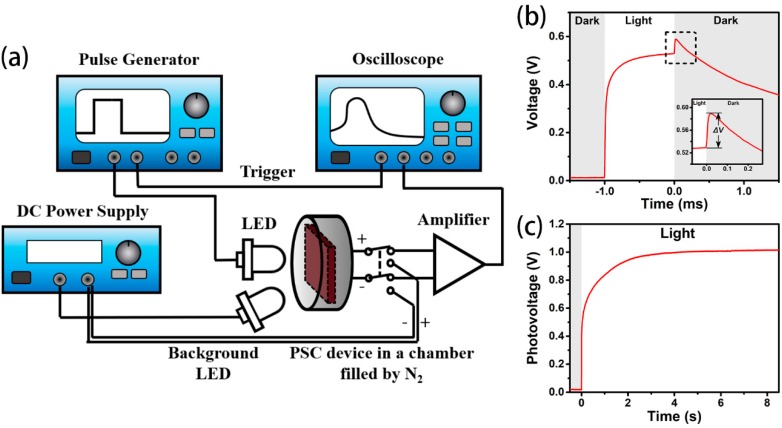
(**a**) Schematic diagram of transient photovoltage (TPV) measurement and (**b**,**c**) time-dependent photovoltage curve of TPV measurement on the PSC device under the illumination of 1-sun-equivalent pulse intensity when the light is (**b**) on at −1 ms and off at 0 ms and (**c**) on at 0 s and off over 9 s.

For the TPV measurement, a pulse generator (WF1946B, NF company, Yokohama, Japan) was used to drive a white light-emitting diode (LED) to generate light pulses with different intensities. The samples were kept in a chamber filled with nitrogen (N_2_) to avoid degradation. The setup for TPV measurement is given in Figure 2a. The TPV signal was taken by an amplifier (OPA1S2384, Texas Instruments, Dallas, TX, USA) with input impedance of 10^13^ Ω. For the TPV measurement under the presence of light bias, another white LED driven by a DC power supply (IT6133B, ITECH, New Taipei City, Taiwan) was used to provide a continuous background illumination as photobias.

Figure 2b shows a typical time-dependent voltage (*V-T*) curve of TPV measurement on the PSC with the structure of TiO_2_/MAPbI_3_/spiro-OMeTAD. The sample was illuminated by a pulsed white LED with 1-sun-equivalent intensity. The width and repetition rate of the light pulse were 1 ms and 1 Hz, respectively. The gray area and white area in Figure 2b indicate light being off and on, respectively. As shown in this figure, the whole *V-T* curve includes four parts: a fast-rising period (~0.1 ms) at the very beginning of the illumination, a slow-rising period in the remaining time of illumination, a fast upshift (overshoot, *ΔV*) after light pulse off, and a continuous descent after the upshift. Herein, our work is focused on investigating the evolution of the *V_oc_* overshoot under different illumination and device pretreatments.

**Figure 3:**
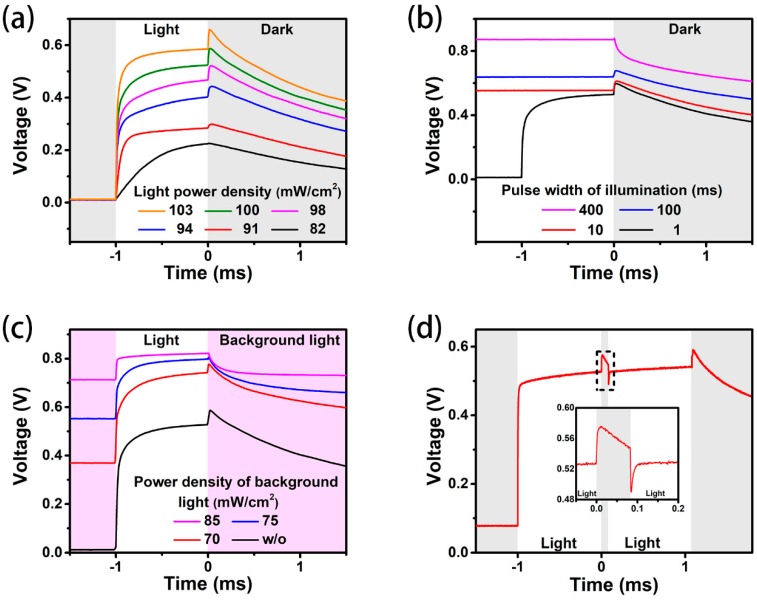
TPV measurement of PSC devices: (**a**) the dependence of TPV on the intensity of light pulses; (**b**) the dependence of TPV on the width of light pulses; (**c**) the TPV obtained under the presence of light bias with different intensities; (**d**) the TPV obtained under the illumination of double light pulses with an 80 μs interval. All curves, except for (**a**), were obtained under the illumination of 1-sun-equivalent pulse intensity. Except (**b**), the light is on at −1 ms and off at 0 ms.

Figure 3a shows the transient *V-T* curves of the PSC device under the illumination light pulses with different intensities. When the intensity of illumination light pulse increased from 82 to 103 mW/cm^2^, the upshift of *V_oc_* increased from 0 to 75 mV. Figure 3b shows the transient *V-T* curves of the PSC under illumination with different pulse widths. The illumination pulse started at −400, −100, −10, and −1 ms. The upshift of *V_oc_* decreased from 67.7 to 8.4 mV along with the increase in the pulse width from 1 to 400 ms. Figure 3c presents the transient *V-T* curves of the PSC device under the presence of a light bias with different intensities. Here, the pink areas indicate that the pulse illumination is off and the light bias is on. It can be observed that when the light intensity increased from 0 to 85 mW/cm^2^, the overshoot decreased from 59.1 to 1.2 mV and nearly disappeared. Figure 3d shows the transient *V-T* curve of the PSC device under the illumination of double light pulses with an 80 μs interval. The *V_oc_* showed a sudden descent of 58.0 mV when the second light pulse was on. The decent of *V_oc_* equaled the overshoot of 58.9 mV when the first light pulse passed through. All the results presented in Figure 3 prove the existence of *V_oc_* overshoot in PSCs with TiO_2_ as ETL.

In general, *V_oc_* originates from the splitting of electron and hole quasi-Fermi energy levels actuated by light illumination:

(1) $$V_{oc}=\frac{1}{q}\left(E_{FN}-E_{FP}\right)$$

where *q* is the elementary charge, and *E_FN_* and *E_FP_* are the electron and hole quasi-Fermi levels, respectively [29]. In TPV measurement, *V_oc_* can be related to the charge density in the device, assuming that the photovoltage is equal to the quasi-Fermi level splitting:

(2) $$V_{oc}=\frac{k_{B}T}{q}ln\left(\frac{np}{n_{0}p_{0}}\right)$$

where *k_B_* is the Boltzmann constant; *T* is the temperature; *q* is the elementary charge; *n* and *p* are the total concentrations of electrons and holes; and *n_o_* and *p_0_* are the intrinsic electron and hole concentrations, respectively [25]. For a device kept under open-circuit condition, no photogenerated charge carriers are produced when the illumination is switched off, and the number of existing electrons and holes keeps decreasing due to the recombination within the device, resulting in a continuous descent of *V_oc_* theoretically. However, the TPV curve of PSC we obtained showed an overshoot of voltage when the illumination was off, which was in contradiction with the above analysis for normal solar cells.

Indeed, under the illumination of the millisecond-timescale light pulse, the photovoltage was different from the *V_oc_* obtained from *J-V* measurement under continuous illumination with the same light intensity. Our time-dependent photovoltage measurements revealed that it took more than 8 s for the photovoltage to reach its maximum *V_oc_* under a continuous illumination of 1-sun-equivalent intensity (Figure 2c). According to the literature, the slow rise of *V_oc_* is associated with ionic migration under illumination [30]. Normally, *V_oc_* is related to the number and the distribution of charge carriers. Nie et al. demonstrated that photogenerated carriers could populate the light-activated trap states under light and relax in dark using photocurrent transient measurements and PL spectra [31].

**Figure 4:**
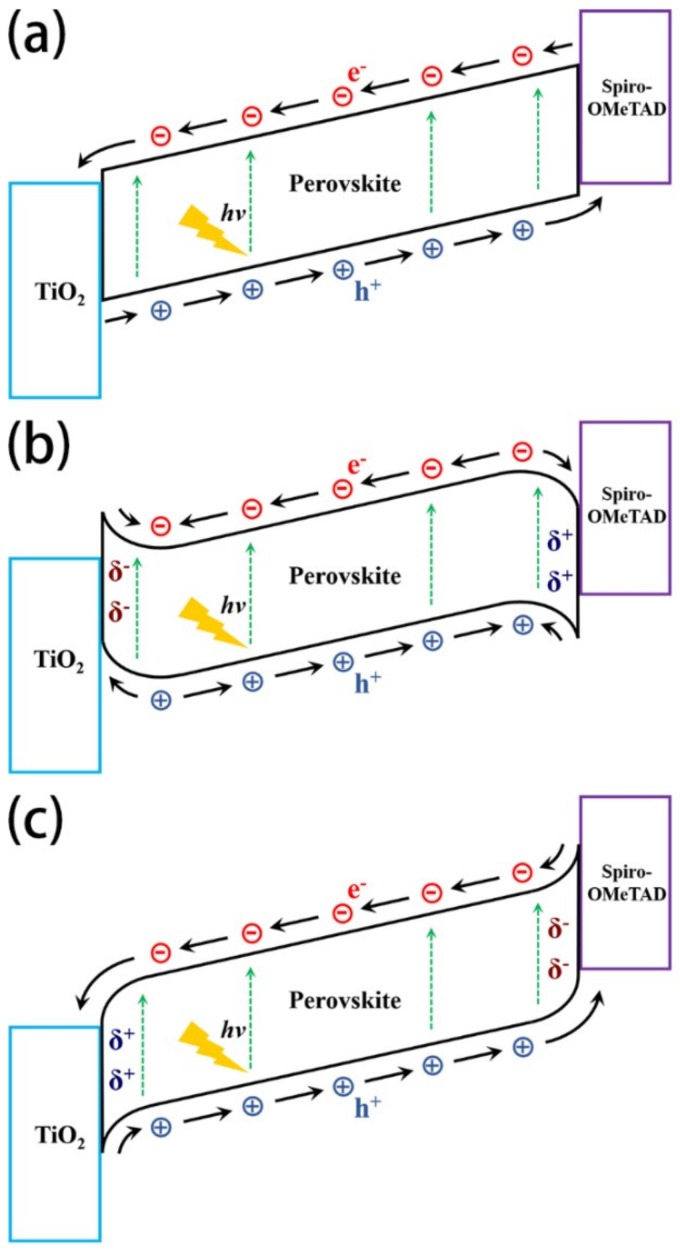
Schematic diagram of PSC device (**a**) without ionic accumulation; (**b**) with anions and cations accumulating at the interfaces of TiO_2_/perovskite and spiro-OMeTAD/perovskite, respectively; (**c**) with cations and anions accumulating at the interfaces of TiO_2_/perovskite and spiro-OMeTAD/perovskite, respectively.

Previous researches have reported that ions do exist and are able to migrate in the MAPbI_3_ perovskite layer [4,18]. Under the drive of the built-in electric field, anions migrate and accumulate at the interface between TiO_2_/perovskite, which would lead to the upward bending of energy band and the formation of barrier for electrons. The same thing also happened at the interface between spiro-OMeTAD/perovskite for holes. The energy level diagrams of PSC without and with ionic accumulation are given in Figure 4. For the PSC without ionic accumulation, all the photogenerated electrons/holes drifted to cathode/anode under the modification of the built-in electric field directing from cathode to anode, except some carriers that recombined via carrier-carrier recombination and defect recombination. When the PSC was applied at a negative bias or a bias less than the built-in potential, the mobile anions were driven to the region close to TiO_2_ electron collection layer, and the cations migrated to spiro-OMeTAD hole collection layer, which led to the energy band bending in both regions (as shown in Figure 4b). Due to the energy band bending, the photogenerated carriers in these regions were driven to the wrong directions with respect to charge collection, leading to an increased accumulation of minority carriers at two perovskite interfaces. This meant that the direction of the electric field in these two regions was opposite to that of the built-in electric field. The inverted electric field reduced the collection efficiency and changed the distribution of the charge carrier, which caused an obvious decrease in *V_oc_*. The intensity of the opposite electric field increased with the number of carriers generated close to the carrier transport layers. Because the light goes through the PSC from the TiO_2_ side, the interface between TiO_2_ and perovskite should contribute more to the inverted electric field. When the light was turned off, no carriers were generated, and inverted electric field disappeared, which led to an enhanced *V_oc_*. Although the number of carriers decreased because of recombination, the redistribution of carriers was much faster than the recombination of carriers. The rising time for the overshoot of *V_oc_* was about 20 μs, which implied that the overshoot was an electronic behavior, instead of an ionic behavior. If PSC was applied at a positive bias larger than the built-in potential, the anions and cations drifted toward to spiro-OMeTAD and TiO_2_, respectively. They accumulated close to the respective interfaces, and the energy bands in the cation and anion accumulation region bent downward and upward, respectively, as shown in Figure 4c. In this situation, the direction of carrier-motion-induced electric field was the same as that of the built-in electric field in the energy band bending regions, which benefited to the carrier collection and resulted in an increase of *V_oc_*. The difference in ionic accumulation is the main reason for hysteresis in PSCs [4].

The overshoot enhanced with the increasing intensity of light pulse, as shown in Figure 3a, because the intense light pulse generated more carriers, which moved to opposite directions, thus enhancing the *V_oc_* overshoot. When the width of the light pulse was increased, more carriers were generated, thus the *V_oc_* became larger. By contrast, the overshoot abated with the increasing width of the light pulse. When the width of the light pulse was 400 ms, the overshoot of *V_oc_* nearly disappeared. With increasing width of the light pulse, more electrons and holes were generated, and the energy valley near the interface of TiO_2_/perovskite was gradually filled with more electrons. Some of them would have got over the barrier caused by ionic accumulation, and the *V_oc_* overshoot became unobvious. The *V-T* curve measured under the illumination of a double light pulse further verified the existence of the reverted electric field. After the first light pulse passed through, the carriers generated by the first light pulse still stayed at conductive band or valence band for hundreds of milliseconds. When the second light pulse was on, the carrier motion against the collection layers turned on the inverted electric field, thus causing a decrease in *V_oc_*.

**Figure 5:**
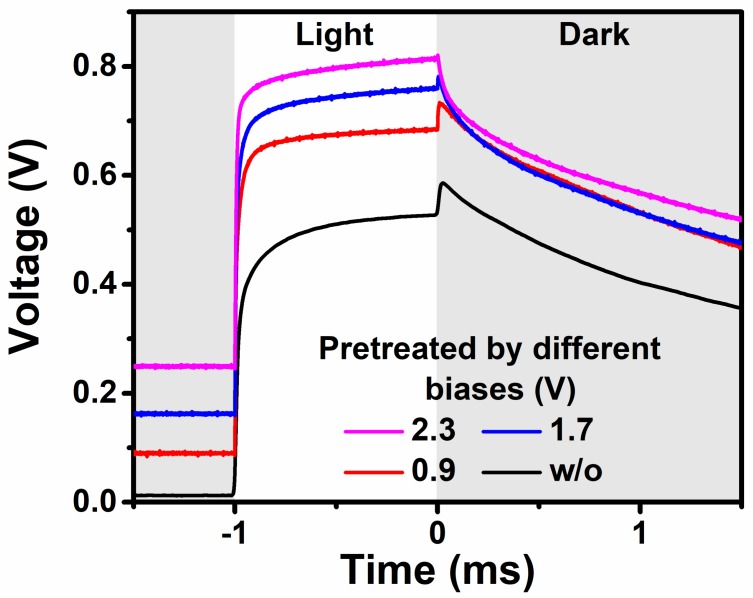
TPV of PSC pretreated with different electric biases for 90 s before TPV measurement.

As previously mentioned, when the PSC is applied at a positive bias higher than the built-in potential, the energy band will bend, as shown in Figure 4c. In this situation, *V_oc_* overshoot should disappear. In order to verify this assumption, the PSCs were pretreated with different positive biases for 90 s before the TPV measurement. When the pretreatment bias increased from 0.9 V to 2.3 V, the overshoot decreased from 59.1 mV to 6.2 mV, as shown in Figure 5. This was direct evidence that the ionic formation induced by the built-in electric field was the origin of the *V_oc_* overshoot.

Weber et al. showed that the formation of ionic charges at the interfaces is the dominating factor for *J-V* hysteresis [32]. Pockett et al. performed a similar TPV measurement at low temperature and attributed the *J-V* hysteresis to ionic redistribution and interfacial recombination [33]. In this study, we changed the starting conditions of the ionic formation and observed the corresponding changes in the *V_oc_* overshoot. These results showed that the *V_oc_* overshoot was related to the starting condition of ionic formation. It is believed that the *V_oc_* overshoot should be associated with hysteresis.

## 4. Conclusions

TPV measurement is an effective technique to investigate PSCs. In this work, we performed this measurement on PSCs, and a *V_oc_* overshoot behavior was observed. The influence of several factors on the *V_oc_* overshoot was investigated, including the pulse illumination density, the pulse width of illumination, the background illumination, and the pretreatment of external electric field. The *V_oc_* overshoot mainly originated from the barrier-induced band bending that originated from ionic accumulation. This work provides a framework to understand the ionic-migration-induced phenomena of PSCs.

## Supplementary Materials

The following are available online at http://www.mdpi.com/1996-1944/11/12/2407/s1, Figure S1: Transient *V-T* curve of organic solar cell with the structure of ITO/TiO_2_/PTB7:PC_71_BM/MoO_3_/Ag under the illumination of 1-sun-equivalent pulse intensity. The light is on at −1 ms and off at 0 ms. Figure S2. SEM image of the surface of perovskite layer. Table S1: Characteristics of PSC device.
