# Supplementary material for: Investigation on the Overshoot of Transient Open-Circuit Voltage in Methylammonium Lead Iodide Perovskite Solar Cells
Source: Materials (Basel). 2018 Nov 29;11(12):2407. doi: 10.3390/ma11122407 (PMC6316959; doi:10.3390/ma11122407)
Supplement: Supplementary file 1 [file materials-11-02407-s001.pdf]

Supplementary Materials

Investigation on the Overshoot of Transient Open-Circuit Voltage in Methylammonium Lead Iodide Perovskite Solar Cells

Chunhai Li, Longfeng Lv, Liang Qin, Lijie Zhu, Feng Teng, Zhidong Lou, Zhenbo Deng, Yufeng Hu, Qiuhong Cui and Yanbing Hou *

Key Laboratory of Luminescence and Optical Information, Ministry of Education, Beijing JiaoTong University, Beijing 100044, China; 12118408@bjtu.edu.cn (C.L.); 11118405@bjtu.edu.cn (L.L.); 14118430@bjtu.edu.cn (L.Q.); 12118410@bjtu.edu.cn (L.Z.); fteng@bjtu.edu.cn (F.T.); zhdlou@bjtu.edu.cn (Z.L.); zbdeng@bjtu.edu.cn (Z.D.); yfhu@bjtu.edu.cn (Y.H.); qiuhcui@bjtu.edu.cn (Q.C.)

* Correspondence: ybhou@bjtu.edu.cn; Tel: +86-010-51684860

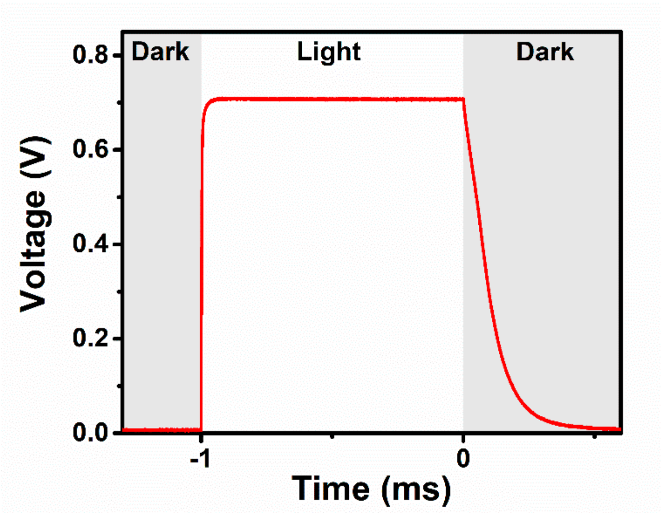

Figure S1. Transient *V-T* curve of organic solar cell with the structure of ITO/TiO_2/PTB7:$PC_{71}BM$/MoO_3/Ag under the illumination of 1-sun-equivalent pulse intensity. The light is on at −1 ms and off at 0 ms.

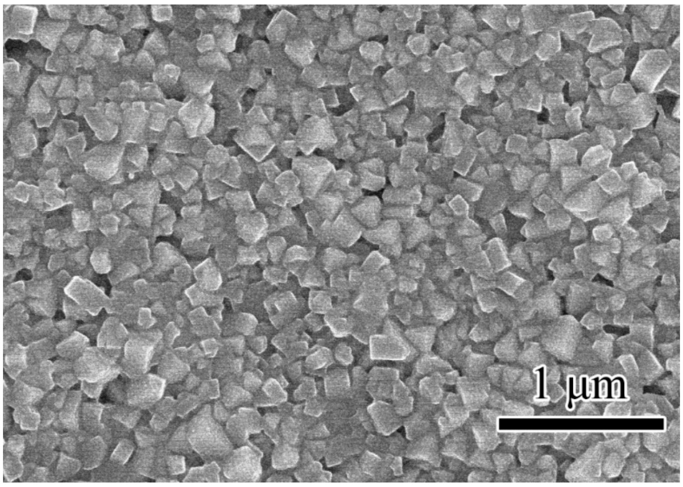

Figure S2. The surface SEM image of perovskite layer.

Table S1. Characteristics of PSC device.

Scanning direction	J_{sc} (mA/cm²)	V_{oc} (V)	FF	PCE (%)
Forward	17.6	0.97	0.52	8.9
Reverse	18.4	1.04	0.71	13.51
